# Patient empowerment: a critical evaluation and prescription for a foundational definition

**DOI:** 10.3389/fpsyg.2024.1473345

**Published:** 2025-01-17

**Authors:** Antonio J. Varela, Michael J. Gallamore, Noah R. Hansen, Dakota C. Martin

**Affiliations:** ^1^Physical Intelligence, Fort Smith, AR, United States; ^2^Arkansas Colleges of Health Education, Fort Smith, AR, United States

**Keywords:** autonomy, behavioral change, self-determination, patient empowerment, resilience, self-efficacy

## Abstract

**Introduction:**

The evolution of healthcare continues to display an incongruence between delivery and outcomes. Current healthcare paradigms for patient empowerment warrants analysis. A lacking operational application for and agree upon assessment of patient empowerment contributes to healthcare’s incongruence. Interchangeable psychosocial concepts and specific patient contextual factors associated with health-related behavioral change have escaped an applicable definition of empowerment. The aim of this theoretical perspective review is to support a comprehensive and contextual understanding of patient empowerment that frames a definition for future consensus research.

**Methods and mechanisms:**

A theoretical perspective review of patient empowerment including interchangeable concepts and patient contextual factors such as personal suffering and resilience; self-determined meaning and purpose; and autonomy, competence, and self-efficacy are critically analyzed. This analysis builds on adjacent concepts including therapeutic alliance, communication, motivation, and trust. The inclusion of specific patient contextual factors that relate to behavioral change elevate the need to reinforce coping and self-management skills as mechanism for patient empowerment. Practice gaps for those experiencing chronic disease, pain, and mental health disorders in rehabilitation setting are specific populations who benefit from healthcare providers unifying the variables associated with patient empowerment.

**Results and discussion:**

The review of associated concepts synthesized an actionable definition of patient empowerment that serves as a foundation for future research. Behavior related changes occur through the evolution in one’s identity, perceptions, and abilities. Interventions that inspire autonomy, competence, and relatedness with a renewed sense of purpose establish resilience and self-efficacy. The totality of this inspired self-determined plan of care establishes the mechanisms required for behavioral change and sustainable transformation. The cumulative experience becomes patient empowerment.

## Introduction

Non-communicable diseases, chronic pain, and mental health disorders represent a growing burden on global health (Hambleton et al., 2023; Connery et al., 2020). The trajectory of these global health burdens correlates directly with levels of disability and inversely with quality of life (Hambleton et al., 2023; Coelho et al., 2009; Garmany et al., 2021; Prynn and Kuper, 2019; Upadhyay, 2022). Management of this global health burden often targets behavioral choices, socioeconomic disadvantages, and environmental factors (Budreviciute et al., 2020; Kelly and Russo, 2018; Manderson and Jewett, 2023). However, healthcare models and policies fail to mobilize behavioral interventions for preventing and managing chronic diseases (Barbosa et al., 2021; Zerwekh, 1992). Changes in healthcare models promoting patient-centered care partially addressed the growing burdens. Patient-centered care was designed to empower patients by promoting personal connections that endorse behavioral change. Patient empowerment has evolved as a concept that allows patients to actively participate in their health-related decision-making processes (Barbosa et al., 2021; Castro et al., 2016; Fumagalli et al., 2015). This evolving healthcare model has increasingly aligned with biomedical models by engaging patients through information and skill-based campaigns to inspire the logical need to change behaviors (Barbosa et al., 2021; Gibson et al., 2021). Attempting to empower patients in this manner has been observed as an adaptive reflection of healthcare’s historical hierarchical model whereas empowerment is something that providers give to their patients (Gibson et al., 2021; Anderson and Funnell, 2010; Halvorsen et al., 2020). Patient-centered care seemingly operationalized the healthcare provider while overlooking the patient’s experiential perspective that often includes a sense of powerlessness (Gibson et al., 2021; Halvorsen et al., 2020; Caston et al., 2024; McAllister et al., 2012). Healthcare models and policies have yet to adopt an authentic provider and patient connection that eclipses traditional assumptions (Gibson et al., 2021; Caston et al., 2024). A more expansive approach that identifies the full expression of empowerment as a process of self-transformation is a worthy endeavor.

The term patient empowerment has become an important concept within healthcare and rehabilitation models that seek to target specific diseases, dysfunction, impairments, risks, and associated disabilities. However, an operational application for and an agreed upon assessment of patient empowerment has no consensus (Barbosa et al., 2021; Fumagalli et al., 2015; McAllister et al., 2012; Mora et al., 2022; Stepanian et al., 2023). Additionally, the relational nature of an individual’s experience remains an elusive factor toward an applicable understanding of self-management and empowerment (Barbosa et al., 2021; Mora et al., 2022; Stepanian et al., 2023; Aujoulat et al., 2008). A consensus defining empowerment inclusive of interchangeable concepts, such as self-efficacy, and respective of the patient’s contextual factors escapes healthcare paradigms. The authors argue that interchangeable concepts and personal contextual factors are variables that act as confounders and modifiers that define patient empowerment and influence patient outcomes (Boers et al., 2014; Robbins et al., 2018).

## Objective

The aim of this manuscript is to support a comprehensive and contextual understanding of empowerment and frame the need for a consensus definition for healthcare providers in rehabilitation settings.

The following objectives will achieve the primary aim:

- Critically review the foundational perspectives and current applications of therapeutic alliance and patient-centered care.
- Provide a comprehensive analysis of specific interchangeable concepts and personal contextual factors.
- Explore how behavioral change is a consequence of empowerment for long-term independence, sustainable function, and improved quality of life.
- Systematically articulate an applicable definition of empowerment that serves as a potential foundation for future consensus research.

A foundational theme for empowerment as a long-term solution for self-management and behavioral adaptations requires insight into variables influencing healthcare providers, patients, and intervention outcomes (Barbosa et al., 2021; Anderson and Funnell, 2010; Caston et al., 2024; Aujoulat et al., 2008; Robbins et al., 2018). Unique contextual factors that will be explored include patient identity (Castro et al., 2016; Fumagalli et al., 2015; Aujoulat et al., 2008); acceptance of personal suffering and gained resilience (Caston et al., 2024; Aujoulat et al., 2008; Phong, 2024); self-determined meaning and purpose (Aujoulat et al., 2008; Ryan and Deci, 2017); autonomy, competence and self-efficacy of regulatory behaviors (Phong, 2024; Ryan and Deci, 2017; Bandura, 1977; Cattaneo and Chapman, 2010); and an energy efficiency toward their goals and intentions (Bandura, 1977; Quigley et al., 2021). Exploring these factors recognizes several adjacent concepts including communication and partnership (Barbosa et al., 2021; Castro et al., 2016; Mora et al., 2022; Halabi et al., 2020; Weisbeck et al., 2019), motivation (Ryan and Deci, 2017; Thomas and Velthouse, 1990), therapeutic alliance and trust (Krupnik, 2023; Zimney, 2020).

**Figure 1:**
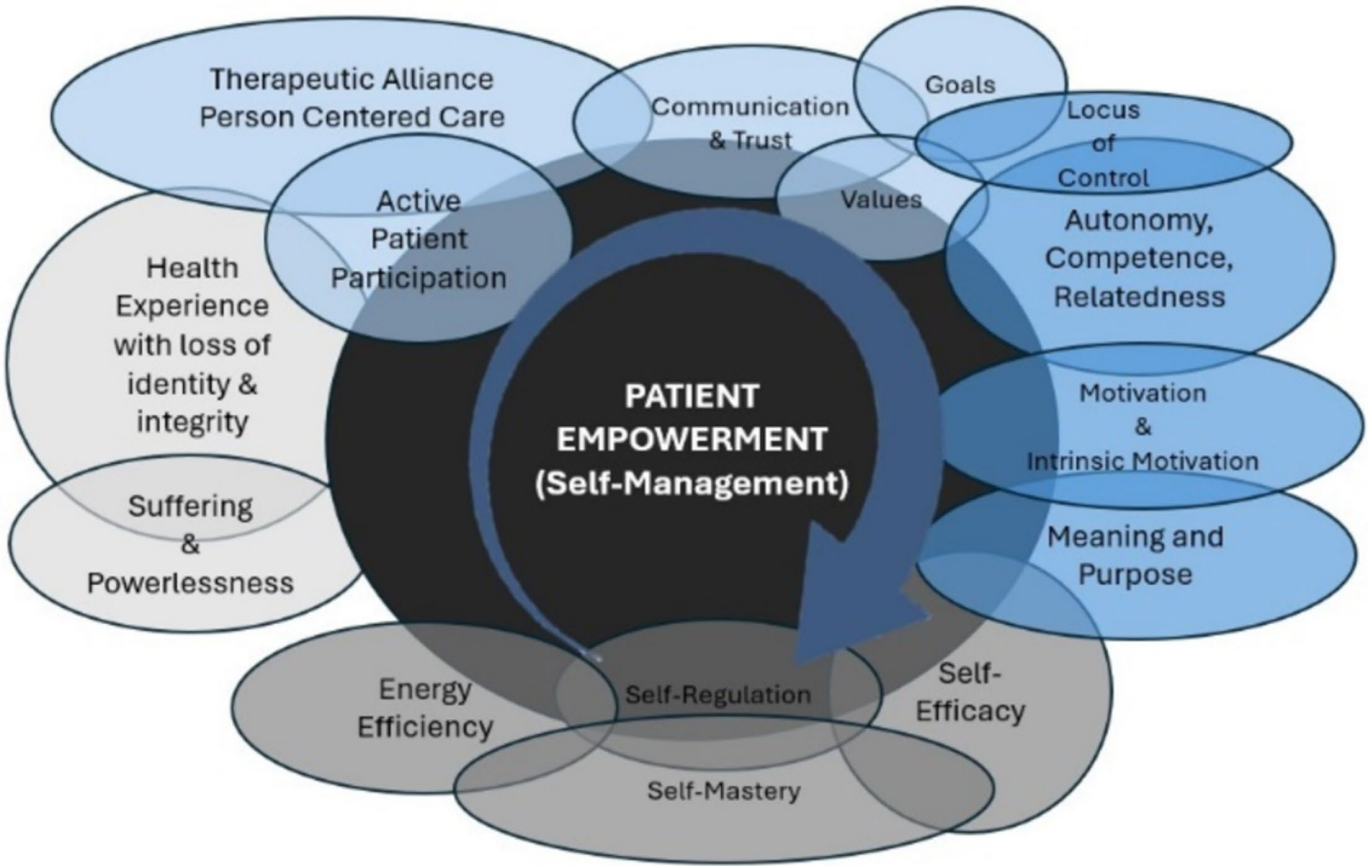
The totality of empowerment: represents the overlapping nature of interchangeable concepts and patient contextual factors that contribute to the dynamic transformation of empowerment. The light grey spaces identify the context from where empowerment is initiated. The light blue spaces identify the antecedents to empowerment. The darker blue spaces identify the attributes of empowerment. The darker grey space identifies the consequences of empowerment. The exception is that self-efficacy is both an attribute and consequence of empowerment. All these concepts and factors will be explored though several will be highlighted as major themes.

Understanding personal and contextual factors including identity, [intrinsic] motivation, experiential suffering, clarified meaning & purpose, resilience, self-efficacy, and consolidated energy that direct internal control and external resources toward self-determined goals are grounding principles that frame empowerment. Several of these concepts such as motivation, self-efficacy, and therapeutic alliance have relatively stable definitions, which are constitutionally adopted to help frame patient empowerment. Synthesizing interchangeable concepts and personal contextual factors represent patient empowerment as a process of self-directed behavioral change with potential to serve future consensus research (Barbosa et al., 2021; Castro et al., 2016; Mora et al., 2022; Stepanian et al., 2023) (see Figure 1).

## Background

### Empowerment

This theoretical review of empowerment focuses on understanding the contextual fluidity of the patient’s experience and the essential processes that enable personal transformation and behavioral change. Healthcare’s adoption of empowerment has become a ubiquitous term representing a patient-centric approach that promotes self-management to produce the most desirable outcomes. However, the extensive utilization of the term without clarified processes specific to the individual has constrained its effectiveness to healthcare’s historical hierarchical model.

A descriptive review identified that 39 different definitions for patient empowerment were inconsistently used (Mora et al., 2022). The definitions of empowerment established general foundational concepts with inconsequential application. These definitions also acknowledged an alternative to compliance-oriented approaches typical to the hierarchical provider-patient relationship (Barbosa et al., 2021; Aujoulat et al., 2008). As such, empowerment also intended to include a patient-centric, collaborative approach that starts with the individuals’ inherent capacity to be in control of their life and facilitate self-directed behavior change (Barbosa et al., 2021; Anderson and Funnell, 2010; Mora et al., 2022; Stepanian et al., 2023). However, a patient-centric approach that supports empowerment as a dichotomous variable with an anticipated outcome still displaces patient’s inherent control to the provider.

Healthcare’s historical hierarchy exemplified power dynamics that provide aid to personal suffering through a pervasive helplessness, powerlessness, and vulnerability. This power dynamic seemed to suggest empowerment is the process of transferring power from one person to another when vulnerability is experienced in an environment of authority and choice (Rodwell, 1996). Empowerment has not evolved past the concept of active patient participation that requires a sense of resilience (Fumagalli et al., 2015; Ungar and Theron, 2020). The conclusion was that patients with a sense of resilience became empowered, and those without represented a dependency on healthcare. Currently, there is no postmodern approach to prioritize the authentic transformation of patient empowerment.

Patient-centered care mirrored by empathy and collaboration that facilitates the co-creation of knowledge are antecedents to patient empowerment (Castro et al., 2016). Further, the patient who enters the healthcare system must be willing to actively participate in their own healthcare (Castro et al., 2016; Mora et al., 2022). Active patient participation is another antecedent to empowerment (Castro et al., 2016). The contextual portrayal of the patient’s experience has escaped healthcare’s understanding of empowerment. This is evidenced by the World Health Organization’s (WHO) definition of empowerment that established four fundamental components: patient participation, patient knowledge, patient skills, and a facilitating environment (World Health Organization, 2023). The WHO’s definition, like other definitions, endorses patient-centered care but lacks contextual experiences and specific processes to follow through.

Empowerments inclusion of authority was juxtaposed by locus of control (McAllister et al., 2012; Zerwekh, 1992). Powerlessness represented the absence of internal locus of control (Richard et al., 2011). Internal locus of control for rehabilitation is a process contingent on personal active choice(s) (Richard et al., 2011). Therefore, the juxtaposition between power, authority, and locus of control revealed a provider’s inherent necessity when helplessness, powerlessness, and vulnerability were experienced by a person seeking aid. This inherent necessity in healthcare could be seen to contain an element of coercion with promotion of behavioral change and self-management (Richard et al., 2011). An individual’s locus of control specific to an ability to be resilient and independently manage must be highlighted, restored, and reinforced as a priority for empowerment.

The context of patient empowerment begins with a healthcare experience that challenges personal identity, integrity, and progress (Caston et al., 2024; Wahlin, 2017). Consequently, the search for the mechanisms of empowerment comes into focus alongside a contiguous exploration of helplessness, powerlessness, and vulnerability experienced through illness, pain, stress, and suffering. The process of empowerment varies according to these contextual experiences and represents the potential mechanisms by which a sense of control is progressively gained or lost. The process of patient empowerment is best established through reflection and self-awareness toward personal identity, experiential suffering, and ability to cope (Castro et al., 2016; Caston et al., 2024; Stepanian et al., 2023; Aujoulat et al., 2008). Personal understanding toward an integrated self is a consequential factor of empowerment (Castro et al., 2016). A patient expresses empowerment as a transformative process rather than an outcome similar to a journey rather than a destination (Anderson and Funnell, 2010).

Patient empowerment is conceptualized as a transformational continuum viewed as movement away from or toward powerlessness. Healthcare providers may circumvent empowerment’s contradiction between authority and powerlessness through a therapeutic alliance that reinforces co-creative and co-constructive power sharing with the specific intention to recognize and build ability, motivation, and self-efficacy toward personal control and self-management (Tengland, 2008). The development of personal control with meaningful capacity to manage everyday life requirements reflects accountable responsibility (Rodwell, 1996). Successful empowerment initiated by a patient’s acceptance of responsibility is noted when a personal sense of powerlessness is alleviated and independence is maximized (Hawks, 1992).

### Therapeutic alliance

Therapeutic alliance (TA) as a bridge to self-management, successful outcomes, sustained recovery, and patient empowerment has inundated research exponentially (Resnik and Jensen, 2003; Horvath, 2018). TA is an antecedent for patient empowerment (Castro et al., 2016). The patient’s fundamental need for validation of stress and suffering opens a personal and genuine exchange that transforms a transactional relationship into an alliance (Caston et al., 2024; Hutting et al., 2022). However, the literature presents a void in the patient’s suffering and contextual powerlessness. The transformative dimensions of empowerment are null and void without an understanding of suffering and personal experiences associated with illness, pain, and disability.

Therapeutic Alliance is an expansion of patient-centered care (a.k.a., person-centered care) that works to transcend the relational nature of personal healthcare experiences. TA and person-centered care (PCC) take a biopsychosocial perspective that focuses on personal and contextual experiences (Hutting et al., 2022). TA and PCC are complementary, whereas the latter helps to forge the former (Holmstrom and Roing, 2010). More accurately, TA is characterized as a patient-centered approach recognizing the whole person while offering a trusting and realistic transference of emotions, traits, and understanding that enables and reinforces self-efficacy, self-management, and empowerment (Castro et al., 2016; Hutting et al., 2022; Krupnik, 2023; Zimney, 2020).

Three specific features are required to formulate TA: an agreement on goals, assignment of responsibilities, and a positively evolving bond (Zimney, 2020; Bordin, 1979; Hauke and Lohr, 2022). These features are similar to those established for PCC, which include a biopsychosocial approach, person focused communication, and the support of self-management that is reinforced through goal setting, coaching to self-management, and evaluating goals and future plans (Hutting et al., 2022). Holmstrom and Roing (2010) offer a review of requirements that describe additional features; understanding the personal meaning of illness, recalling the provider perspective is subjective, and specifically recommending prevention and health promotion (Holmstrom and Roing, 2010). The TA and PCC represent an authentic relationship of congruency supported by trust that inspires self-efficacious autonomy and competence with goal setting and accomplishment (Krupnik, 2023; Holmstrom and Roing, 2010; Langer, 1999; Unsgaard-Tøndel and Søderstrøm, 2021).

The healthcare provider’s role is a steward of the patient’s success that empowers the achievement and progression of agreed upon goals. Stewardship implies that the patient has shared a portion of their health custody with their provider. In this stewardship, the provider uses clinical judgment based on the patient’s needs with the best available evidence to facilitate the patient’s recovery (Hutting et al., 2022). The provider collaborates to transfer knowledge to the patient, so heath custody is regained with a sense of autonomy and competency. This transformation encompasses self-management and patient empowerment. However, collaboration only happens through TA that endorses authentic communication, gained trust, and personal goals while validating contextual powerlessness and personal suffering.

### Communication

Another antecedent to empowerment includes effective communication that emphasizes an empathic dialogue that builds trust within the provider-patient relationship (Castro et al., 2016; Krupnik, 2023; Zimney, 2020). The link between a patient’s responsibility to the diagnosis, the treatment plan, and the long-term management of any illness, pain, and disability is established through collaborative communication. Communication must be tailored and comprehensible at a patient-centered level to develop a personal connection with a sense of trust (de Haes and Bensing, 2009). The bilaterality required for communication includes open-ended questions about the individual rather than their medical situation, active listening of their story, and terminology befitting their educational level (Baum, 2023).

Authentic information gathering and information provision are two proposed functions crucial to communication that differ from other research models (de Haes and Bensing, 2009). Information gathering in this context functions by getting to know the patient rather than reducing them to the condition or dysfunction, typical of the biomedical model. The information gathered creates the specificity of an individualized plan. Information provision is a detailed understanding of the patient’s knowledge that tailors and directs information to their cognitive abilities. The provisions of information engage the patient by expanding their accountability, health literacy, and self-efficacy while simultaneously limiting uncertainty. Both functions create patient satisfaction with a sense of empowerment that completes individualized communication through flexible and adaptive PCC (Ricci et al., 2022).

The keystone characteristic of understanding health-related concerns is health literacy. Health literacy marked by effective communication of health concerns is an indicator of empowerment (Bravo et al., 2015). Nutbeam (2000) described health literacy as “a set of cognitive and social skills related to health decision-making.” A patient that lacks the mechanism to effectively understand and communicate their main health-related concerns are experiencing a sense of powerlessness (Schulz and Nakamoto, 2011). Likewise, a patient with high health literacy is or becomes an effective manager of their own health. Another indicator of empowerment includes immediate and long-term outcomes gained from effective medical communication (Bravo et al., 2015). Effective communication serves as a conduit for the transfer and co-creation of knowledge, which provide self-management and coping skills that empower the patient.

### Trust

Trust has been identified as the most critical feature related to TA, which develops on a continuum of genuine and humble interactions (Krupnik, 2023; Charmant et al., 2021). Genuine interactions that build trust are conveyed through compassion and humility (Krupnik, 2023), the sharing of power (Fugelli, 2001), and the fulfillment of obligations that work in the patient’s best interest (Horner, 2020). Trust is formed by communication that conveys the patient experiences, identity, and perceptions were validated (Caston et al., 2024; Langer, 1999; Fugelli, 2001). Additionally, trust is witnessed when a patient accepts the situational risk associated with a level of powerlessness (Luhmann, 2000). However, the features of TA and PCC do not fully account for trust. For example, goal focused communication is inadequate for building trust (Crom et al., 2020). The bond formed through trust is an attribute of empowerment that continues to facilitate personal growth (Castro et al., 2016; Caston et al., 2024). The bond is preserved by the healthcare provider’s humble commitment to validating the patient (Fugelli, 2001; Horner, 2020).

Providers must also recognize how patients who have experienced a betrayal of trust by the healthcare system become vigilant in their defensiveness when placed in further vulnerable situations. Epistemic vigilance derived from a negative experience such as medical discounting and neglect become barriers to TA (Krupnik, 2023). Consequently, apprehension to trust disrupts the processing of information and ultimately blocks the development of TA causing the patient to persistently defend themselves against vulnerability (Fonagy and Campbell, 2023). Therapeutic interaction requires a validation process that is reliant on life experiences (Caston et al., 2024; Venta, 2020). The effective provider-patient relationship will have a balance between trust and epistemic vigilance. Lastly, the ability to recognize the patient’s epistemic vigilance and defensiveness to vulnerable communication offers opportunity to foster TA built on trust.

#### Relational trust

Relational trust encompasses the working provider-patient relationship established through an authentic relatability. The ability to relate is grounded in an empathic response to the individual and their helplessness, powerlessness, and vulnerability. There are two main processes of empathy: understanding and response (Brock and Salinsky, 1993). Empathy is a precursor to the validation of the patient’s feelings and is vital for the recognition of their suffering, which dissolves epistemic vigilance. Alleviation of epistemic vigilance occurs through a relational trust that allows the patient to recognize a power dynamic while fostering autonomy by leveling the top-down approach. Studies described relational trust as a mutual interaction and connection with acceptance and respect with co-ownership of decisions (Vestol et al., 2020). This mutual interaction alleviates the hierarchal divide and reinforces the relational trust and initiates a path toward empowerment.

#### Co-creation of knowledge

High quality provider-patient interactions built on trust unlock sources of awareness that allow patients to openly accept new knowledge. Aujoulat et al. (2008) offered practical applications for empowerment by redefining the provider-patient relationship, emphasizing the importance of collaborative dialogue unique to a patient’s life experiences. Collaboration enables the co-creation of knowledge through engaging dialogue, idea testing, problem-solving, and developing value orientation. This intellectual growth facilitates cognitive flexibility, problem-solving skills, and inspires self-determination toward behavioral awareness and change (Caneiro et al., 2021). Cognitive Behavioral Therapy exemplifies this collaborative dialogue whereas the provider and patient work together to identify maladaptive tendencies and recognize personal values that reorient behavior. The consequence of patient participation in co-creating knowledge is the promotion of autonomy, competence, intrinsic motivation, and self-efficacy that dissolves initial powerlessness (Castro et al., 2016; Weinstein and Ryan, 2011).

A collaborative relationship within a nurturing environment that provides evidence-based choices and ideas reflective of personal experiences and values empowers a patient to derive their own preferences. Co-creative knowledge that reorients personal values and redirects experiential factors will reinforce the self-transformation toward personal control of individualized expectations and outcomes. Empowerment is ultimately seen as self-determined, self-efficacious, and self-regulated management gained through interpersonal education and intrapersonal awareness of coping strategies and effective treatments for their illness, pain, and disability management (Unsgaard-Tøndel and Søderstrøm, 2021).

### Suffering

Healthcare’s paradigms have neither defined nor embraced the context of patient suffering. Healthcare providers must acknowledge, account for, and explore suffering if the full experience of health and life is to be fully understood. Providers continue to struggle addressing the healthcare related frustrations that turn the patient’s identity, perceptions, and life experiences upside down (Caston et al., 2024). Existential frustration, a term coined by Frankl (1966) described a personal sense of meaninglessness and emptiness, may be the initial clinical definition of suffering. The manifestation of existential frustration is either the loss or misdirection of personal identity and purpose in life. Displaced purpose with an experienced emptiness relates directly to a loss of self, a form of suffering that can be endured as depression (Cassell, 1998; Cowden et al., 2022; Vander Weele, 2019).

Frankl (1966) emphasizes that unavoidable suffering is a fact of life inherent to the human experience that should not be denied. Suffering is an experience that enables empowerment and transcendence. Suffering can be defined as the state of distress associated with events that threaten the integrity of the person with a perceived impending destruction (Cassell, 1998). This state of distress directly correlates with insufficient or misunderstood explanation for the destructive experience. The margin of hope between personal challenges and empowerment widens in tandem with a threatening experience associated with a loss of purpose. Great suffering frequents loss of hope (Ross, 1995). Suffering continues until the threat of destruction has passed or until the integrity of the person has been restored (Cassell, 1998; Dedeli and Kaptan, 2013). This transformational concept might consolidate the degree of suffering and introduce the psychological meaning of pain (Bustan et al., 2015).

Suffering is most often linked with pain. People in pain frequently report suffering when they feel it to be chronic, dire, out of control, or overwhelming (Cassell, 1998; Trachsel et al., 2019). Plato defined pain as an emotional experience associated with a perceived penalty that occurs when intense noxious stimulus persists (Kumar and Elavarasi, 2016; Seth and de Gray, 2016). Currently, pain is defined as an unpleasant sensory and emotional experience associated with or resembling that associated with actual or potential tissue damage (International Association for the Study of Pain, 2020). Tissue damage can be perceived as a mechanical destruction associated with loss of personal and functional integrity. The degree of suffering occurs in magnitude to the perception of loss of any aspect of personal integrity including though not limited to body awareness, functional independence, group identification, family dynamics, personal self-efficacy, social roles, or the relation with meaning (Cassell, 1998; Dedeli and Kaptan, 2013).

Pain and suffering are not the same. Pain is the perception of noxious sensory stimulus while suffering is a perception of pervasive personal destruction (Cowden et al., 2022; Fishman, 1992). The two are separated by neural pathways that appraise sensory-discriminative and affective-evaluative dimensions of pain (Bustan et al., 2015). The phenomenological differences in pain and suffering are expressions experienced through different neural pathways (De Ridder and de Wit, 2006; Hart et al., 2003). Chronic pain with persistent stressors create neuroplastic adaptations between the different neural pathways resulting in overlapping interpretations of pain and suffering. Deficient coping strategies reinforce the neuroplastic changes in these pathways (Phong, 2024; Quigley et al., 2021; Hart et al., 2003). The chronicity of pain distorts the processing of the representational self (Hart et al., 2003; Baliki et al., 2008; Broyd et al., 2009; Pei et al., 2020). Pain potentially consumes the individual thereby intensifying the overwhelming and out of control feelings, experienced as suffering (Apkarian et al., 2011).

The relational meaning in which people experience illness, pain, and disability is an essential context to understanding suffering. Illness, pain, and disability perceived as a destruction to the self becomes a pathway for suffering (Apkarian et al., 2005, 2011). This suffering may lead to further counterintuitive adaptations including anger, anxiety, depression, fear, frustration, and disability convictions. Perceptions, emotions, and behaviors reflect the coupling of the brain’s pain and emotional pathways (Apkarian et al., 2011; Edwards et al., 2016). These perceptions, emotions, and behaviors provide insight into the therapeutic mechanism a provider must lean into to extinguish or transcend suffering.

Illness, pain, and disability are individual and universal experiences that invariably cause suffering. These experiences are tangible descriptions of Viktor Frankl’s emphasis on unavoidable suffering. Frankl’s message was that human experience has transcendent potential. The emotional overlay and strain of the experience underscore the message (Bustan et al., 2015). Everyone has the potential transcendent dimension enveloped within their unavoidable suffering. Enduring suffering from the loss of personal integrity may serve as an adaptive function that leads to post traumatic growth (Vander Weele, 2019; Walsh, 2007). The endured suffering produces a mental focus that elevates what is of greatest importance, leads to developing practical inspiration, and forges resilience (Vander Weele, 2019).

The context of suffering is an individual experience that affects the biopsychosocial and spiritual perspectives of the whole person (Apkarian et al., 2011; Snyder and Lopez, 2001). This context makes suffering the experience as opposed to the problem. Therefore, suffering becomes the vector that enables transcendence as it cultivates personal meaning and purpose to fully engage life (Walsh, 2007). Existential clarification with a re-established purpose that is appropriately channeled can be the ultimate result of suffering. This existential clarification also has the potential to activate empowerment.

The experience of loss, illness, disability, or pain can bring someone closer to a valued goal, and that person may then have no sense of suffering at all but rather feel triumph. The triumph of suffering is most directly expressed in religion, whereas suffering is seen as the pathway to understanding God (Sabry and Vohra, 2013). This “function” of suffering is its glorification and its relief (Cassell, 1998). Regardless, personal challenges and the associated emotions that bring them to the precipice must first be accepted (Ballantyne and Sullivan, 2015). Patients who come to understand themselves in response to illness, pain, and disability may grow instead of being reduced. Empowerment through gained clarification of personal identity transforms the awareness of values, goals, and resources, which manifest intrinsic locus of control, motivation, resilience, and self-efficacy (Walsh, 2007; Tedeschi and Calhoun, 2014).

Gaining resilience toward empowerment and overcoming suffering often requires help (Lotz, 2016). Empowerment to challenge adversity must be channeled through the endurance of suffering by the individual even though a portion of that endurance might be shouldered by family, friends, and healthcare providers (Frankl, 1966; Tedeschi and Calhoun, 2014). Healthcare providers can no longer ignore the whole biopsychosocial makeup of the person including suffering. Suffering illuminates a pathway to empowerment gained through TA and collaboration that reconstructs one’s own meaning in life and purpose.

### Meaning and purpose

A critical attribute of empowerment is personal meaning, which directs a patient toward their specific aim. The transformation of suffering may come from a reconstructed attitude towards universal meaning and purpose in life (Hemberg, 2017). Frankl (1966) clarified that meaning in life gives rise to purpose, and the combination is a fundamental need that must be personally discovered. Purpose refers to the intention to form positive meaning from adverse life experiences (Martela and Steger, 2016; Schaefer et al., 2013). Purpose is further defined as a perception that one’s life has core goals, future-oriented aims, and specific direction (Martela and Steger, 2016; Hill et al., 2022; Reker et al., 1987). Purposeful direction, intensity, and duration of action are determined by personal values and goals (Schippers and Ziegler, 2019). Alignment with personal values and goals further distinguishes internal locus of control and motivation required when adversity is experienced (Kang et al., 2019; Ryan and Deci, 2000).

Purpose in life correlates directly to emotional recovery (Hill et al., 2022), which affects physical well-being (Hirooka et al., 2021), activity engagement (Yemiscigil and Vlaev, 2021), and internal conflict in decision-making (Kang et al., 2019). A sense of purpose allows patients to change the perception or interpretation of adverse events that may otherwise cause anxiety and stress (Hill et al., 2022). This positive change of perception or interpretation enables an ability to process events as meaningful, necessary, and valuable to fulfill one’s purpose. An individual’s purpose can be found by exploring three overlapping reflections; personal goals and values, personal fears and anxieties, and coherence of challenging unpredictable circumstances (Wong, 2013). Purpose with a sense of coherence and a feeling of significance sustain meaning in life that best serve overcoming adversity (Kang et al., 2019). Investigating and reflecting upon discovering core values, inhibiting fears, and efficient processing with coherence facilitates self-determination.

Meaningful and purposeful directional pursuits in life require self-reflection and self-regulation. Kang et al. (2019) illustrated self-reflection as the “how” versus the “why” when aligned with purpose. The purpose is the reason “why,” which allows a focus on “how.” Instead of focusing on “why” the adversity happened, the individual can focus on the “how” to overcome adversity. The “why” is an articulation of purpose and is intimately associated with resilience. Absence or disruption in purpose is disempowering and exemplifies an incongruence between values, goals, and outcomes. This incongruence amplifies stressors, promotes suffering, and removes the ability for adaptation to adversity.

A disconnection from meaning and purpose with a loss of psychosocial resources, which includes values and goals, is a unique predictor of stress outcomes (Edwards et al., 2016; Bonanno et al., 2007). Purpose and resilience have a role in mitigating the duration of compromised psychosocial resources required for problem-solving, realignment, and restoration of values and goals. The concept of resilience and resources are associated with purpose. The importance of resilience in this context is the self-regulatory processes that re-establish coherence of adverse events. A patient’s misfortune offers potential to find reinforcement of their purpose and a reorganization of resources that rebuild coherence from shattered assumptions (Park, 2010). Likewise, understanding personal resources helps the comprehension of unpredicted experiences resulting in plan formulation that directs energies toward the achievement of valued goals. Purpose provides a patient with efficient self-organization toward goals, self-regulation for coherence, and self-efficacy to channel resources.

Self-awareness and exploration of values may be a more engaging way to initiate and sustain the journey toward meaning and purpose (Liddiard et al., 2019). A patient’s purpose challenged by unpredictable and adverse events provides salient decision and sense making, which reinforces the meaning of their life. Finding purpose is often a life pursuit, and the pursuit is an exercise in endurance. The journey toward discovery is perhaps the best that can be offered to patients.

### Resilience

The term “resilience,” like empowerment, is considered a process and an outcome. Resilience as a deliberate process leads to a successful adaptation to adversity and as an outcome demonstrates a personal characteristic. An adverse event, a stressor, or suffering are prerequisites for resilience with the potential to forge adaptation. A comprehensive definition of resilience is summarized from interchangeable concepts like empowerment. Resilience is a dynamic, reintegrative process that becomes a stable trajectory toward healthy functioning through a conscious choice and effort to move forward utilizing insight from lessons learned that establish successful adaptations to an adverse event associated with the disruption of personal integrity (Southwick et al., 2014). This definition of resilience subtly integrates the concept of suffering and suggests endurance is required. A long-term recovery and sustainable trajectory are reinforced with one’s ability to harness internal and external resources that sustain well-being (Southwick et al., 2014).

Resilience reflects a patient’s capacity to overcome suffering, which is associated with a commitment to risk tolerance and coping mechanisms (Ungar and Theron, 2020; Baumeister et al., 2007). Capacity for immediate adversity also requires endurance for long-term recovery. Therefore, recovery and sustainability become two different states of resilience. Resilience enables recovery that may or may not align with the individual’s baseline. However, sustainable recovery tilts the trajectory toward a higher plane of existence, which includes mental, physical, and social health (Ryff and Singer, 1998). The difference in sustainable upward trajectories may be noted through an individual’s realignment with valued pursuits that provide purpose and meaning in life beyond recovery (Ryff and Singer, 1998; McKnight and Kashdan, 2009; Lyubomirsky et al., 2005).

Mobilizing and sustaining resilience is clearly marked by the patient’s biopsychosocial makeup. Any clear relationship between the uniqueness of a patient and the complexity of resilience is elusive and difficult to understand. Literature exploring resilience posits that self-regulating capacities and cognitive coping strategies are unsustainable unless other co-occurring physical and social systems (Ungar and Theron, 2020) such as exercise (Arida and Teixeira-Machado, 2021; Deuster and Silverman, 2013; Ho et al., 2015), family (Ungar and Theron, 2020; Hassani et al., 2017), mindfulness (Garland et al., 2015), safety (Hofrath, 2021; McCauley et al., 2012), and spirituality (Duran, 2019) are available enough to support adaptive behavior. The connectedness to oneself and to those providing support along with a sense of higher purpose may exemplify a biopsychosocial approach that unlocks the key to resilience and empowerment (Weinstein and Ryan, 2011; Spake and Thompson, 2013).

Sustainable resilience further implies that recovery setbacks or new challenges are tolerated to a better degree than previous experience. Resilience becomes a defense mechanism against additional adversity and stress. Awareness of protective factors for mental wellbeing dates back to the early 19th century concept of mental immunity, which defined “the art of preserving the mind against all incidents and influences calculated to deteriorate its qualities, impair its energies, or derange its movements” and include “the management of the bodily powers to exercise, rest, food, clothing and climate, the laws of breeding, the government of the passions, the sympathy with current emotions …”(Rossi, 1962, p. 80) The gained immunity related to the characteristic of resilience must have some depth and breadth to specific underlying personal experiential factors.

### Motivation

An individual’s inherent choice initiated by self-determined personal reflection and willingness are characteristics fueling the driving force that directs and sustains behaviors. This underlying driving force is also known as motivation, which is observed through behavior. The concept of empowerment may be popular because it gives shape to the nontraditional concept of motivation (Thomas and Velthouse, 1990). The term motivation is derived from the Latin verb *movere* meaning ‘to move.’ Movement, in this context, falls short of fully defining the term motivation, which involves self-awareness and engagement through goal setting, choice, effort, endurance, persistence, purpose, reason, and regulation to pursue action (Fumagalli et al., 2015; Dornyei and Ushioda, 2021). Motivation requires self-regulation for its distinct role in the pursuit of a treatment outcomes when barriers challenge long-term goals. Self-regulation is critical to personal intention and strategy for consistent follow through with directed action and skill.

An understanding of motivation requires embracing individualistic perspectives associated with personal attitudes, beliefs, preferences, perceptions, traits, and values, which are all framed within their social context (Dornyei and Ushioda, 2021; Lai, 2011). Motivational disposition provides the evidence of a patient’s values as expressed through their thoughts, feelings, and actions (Dornyei and Ushioda, 2021). These personal distinctions, most notably personal values, are tethered to the energy supplying motivation and can predict a tendency to pursue long-term goals (Lai, 2011; Von Culin et al., 2014). Personal values that align with a sense of purpose will funnel motivation into personal and intrinsic motivation (Dornyei and Ushioda, 2021).

#### Intrinsic motivation

Intrinsic motivation is considered an antecedent and an attribute to empowerment (Fumagalli et al., 2015; Halvorsen et al., 2020; Thomas and Velthouse, 1990). Empowerment and Intrinsic motivation are linked by a patient’s inner directedness and self-determination. According to several authors, empowerment and intrinsic motivation share the same four (4) dimensions: meaning, competence, choice, and impact (Thomas and Velthouse, 1990; Spreitzer, 1995). The four dimensions manifest intention toward a specific aim or task (Bandura, 1989). In many ways, intrinsic motivations distinction between developing and executing required skills for goal attainment enforces empowerment.

Intrinsic motivation relies on the balance of personal factors, which influence all tasks regardless of the outcome. More challenging tasks adapt a cyclical expression of the balance between task difficulty, skill set, and execution without concern for the outcome (Landhauber and Keller, 2012). Intrinsic motivation is separated from empowerment through the rewarding experience of a challenging task (Fumagalli et al., 2015). Intrinsic motivation’s process of negotiating task requirements on the fringes of skill set is the reinforcing reward. A specific outcome is the realization of empowerment. Intrinsic motivation’s outcome of a pursuit is predetermined by the perception of competence and level of self-efficacy. In other words, intrinsic motivation provides a focus on the journey rather than the destination.

Intrinsic motivation for a specific pursuit requires a transformative commitment with persistent renewal of effort (Di Domenico and Ryan, 2017). The renewal of effort for a challenge has an inherent reward built within that only requires mindful ownership and grounded presence. Mindful ownership can be viewed through the lens of autonomy, competence, and control, which have been identified as the mediators of intrinsic motivation (Deci and Ryan, 2000). The development of a new skill advances competence but only after acceptance of the existing void in knowledge. Competency begins as an interactive change that builds capacity with some application of new skills (Howland and McGuire, 1968). Advances in perceived competence is the beginning of sustainable intrinsic motivation (Deci and Ryan, 1980).

Self-determination that leads to intrinsic motivation provides the foundation to build competence, which fosters adaptation (Deci and Ryan, 1980). Competency is perpetuated upon successful completion of valued tasks further enabling acceptance of other meaningful tasks or challenges. This perpetuation requires persistent motivation, which is a reconfirmation of active and independent decisions even in the void of positive reinforcement or a conducive environment (Deci and Ryan, 1980). Intrinsically gained motivation and gained competence are derived from personal meaning and purpose and reflect personal choice (DeCharms, 1968).

Established self-determination, intrinsic motivation, and gained competency further develops personal autonomy and locus of control. The perception of internal locus of control lends itself to initiating intrinsic motivation, described here as a requirement for empowerment. Autonomy begins with the self-actualization of one’s decisions and behaviors without external pressures (Di Domenico and Ryan, 2017; Sweet et al., 2012). Further, autonomy and locus of control are conceptually congruent with the ability to interact and control one’s environment toward a specific outcome (Kidd, 2016). Autonomy, competence, and locus of control also serve as mediators to the development of self-efficacy that further fuel intrinsic motivation (Deci and Ryan, 1980; Sweet et al., 2012; Ajzen, 2002). Therefore, the authors argue that intrinsic motivation with outcome expectancy is required before self-efficacy is gained (Sweet et al., 2012).

### Energy efficiency

Empowerment requires energy to fuel the purposeful drive toward valued goals, and their achievement may be traced back to the efficiency of behavior. Behavioral adaptations and responses require efficient storage and utilization of energy. Successful management of personal energy is an attribute for adaptation and integration with the environment (La Cerra and Bingham, 2022). Energy efficiency underscored by a sense of mastery is an attribute of empowerment. Moreover, efficient self-organization and energy budgeting with functional integration is a consequence of empowerment. Ultimately, adapting and surviving illness, pain, and disability are dependent on a dynamic energy budgeting system that regulates interaction with a changing environment.

Energy efficiency as an imperative to survival reflects successful empowerment. The quality of personal empowerment is a multi-dimensional phenomenon coordinated with management of unpredictable circumstances and available resources. Survival equals adaptation and commands personal perspectives to expand, which implements organization. A functionally adaptive patient experiencing situational distress has potential to evolve, reconceptualize, and demonstrate higher order thinking (Marks-Tarlow, 1999). In other words, errors in predicted expectations resulting in distress can result in updated predictions (Godinic et al., 2020). Investment in updated predictions increases self-organized performance, conserves energy, and provides a return on work effort. The efficient utilization of situational distress requires problem-solving and reasoning skills, which is essential for personal growth. The self-organizing patient conserves free energy required to adapt to ongoing unpredictable adversity. Moreover, a self-determined and self-organizing patient can exert change in themselves and their environment through actions based on accurate perceptual judgements and predictions (Bandura, 1989).

Accurate perceptual awareness is required for learning, which is exemplified through adaptation and change while displaying energy efficiency (Friston, 2003). Energy efficiency supplements improving skills. Behind all adaptations is the constancy of distress, which leads to resilience. Resilience is noted by improving adaptive skills and coherence, mentioned in the ‘purpose’ section, that further represents a sense of purpose and efficient cognitive and physical efforts required for psychological and physical well-being. Energy efficiency reinforces an individual’s self-efficacy representing actionable self-determination.

Psychological well-being depends on coherence between subjective and objective awareness, which lends themselves to accurate perceptions and predictions (Hauke and Lohr, 2022). A self-organizing person that fails to establish this coherence displays inaccurate perceptions and ineffectively manages unpredictable circumstances resulting in cognitive mal-alignment and avoidant reactions (Hauke and Lohr, 2022). Prediction errors reveal inefficient energy expenditure. This patient may avoid further self-management with the unrealized intention to conserve energy. The conservation of energy becomes the avoidance of inefficient action likened to fatigue driven passivity. The coping strategy of avoidance with passive responsiveness occurs with a sense of helplessness. Growing helplessness is a weakening of resilience associated with a compromise in behavioral change (Stephan et al., 2016).

Health-related experiences fracture personal expectations and foundations, which create insecurity, instability, and suffering that consume resources and further contribute to the psychological strain and weakening of resilience. The mobilization of available energy becomes adaptive with efficient coping, reflection, and regulation (Weinstein and Ryan, 2011). A well and healthy self-determined patient acts to preserve and maximize their functional integrity in the face of personal adversities, barriers, and challenges. Gaining autonomy and competence to manage incoming adversities with regulated subjective awareness and accurate perception conserves energy while enhancing adaptive behavioral responses.

### Self-efficacy

Self-efficacy is strongly related to the competence dimension of empowerment (Nafradi et al., 2017). In fact, the relationship between patient empowerment and self-management behavior is mediated by self-efficacy (Wang et al., 2022). The construct of self-efficacy was born from biopsychosocial, social cognitive, and self-determination theories. Self-efficacy is defined as a belief in one’s capabilities and confidence to organize and execute a course of action required to manage a prospective situation (Bandura, 1977; Tengland, 2008). Self-efficacy is task dependent and oriented by personal beliefs and expectations. The execution of personal beliefs and expectations are associated with a higher internal locus of control (Keedy et al., 2014). An internal locus of control establishes the outcome expectancy while self-efficacy mobilizes specific skills to accomplish the expected outcome. An individual’s constitution established through self-efficacy may provide the most influential understanding of psychosocial considerations, particularly related to health behaviors (Tengland, 2008; Keedy et al., 2014). As such, measuring self-efficacy through specific questionnaires (i.e., The Empowerment Scale) helps to define personal empowerment (Castro et al., 2016).

Self-regulation, as it relates to health behaviors, is often an overlooked concept within self-efficacy. The importance of self-regulation is the requirement for adaptive behavior and general well-being, needed for recovery and rehabilitation. Self-regulation as a reflection of behavior, energy conservation, and predictor of autonomy refers to efforts that alter thoughts, perceptions, emotions, and actions relative to personal goals (Hart et al., 2003; Carver and Scheier, 2001). Therefore, self-regulation holds patients accountable and responsible to their expectations specific to chosen actions. The relationship between expectations, self-efficacy, and self-regulation orients the individual to a predicted action and reveals behavior (Bandura, 1977). Self-efficacy is observed through efficient behavior exemplifying competence in personal beliefs as seen through effective task completion. The emphasis of self-efficacy is on an individual’s beliefs regarding personal ability to successfully meet situational demands, which explains how and why people either orchestrate or fail to orchestrate specific actions toward the pursuit of an outcome (Bandura, 1995).

Self-determination theory exemplifies an individual’s assessment of their internal and external resources to cope and facilitate a ‘competence of control’ that supports personal self-efficacy (Ryan and Deci, 2017; Bandura, 1977; Sweet et al., 2012). Self-determination theory accounts for the self-organizing capacity to be effective and includes self-awareness, active learning, actionable informed decisions, internalized motivation, and regulation of values (Ryan et al., 2021). The constructs between self-determination theory and self-efficacy can be integrated to enable motivation by harnessing competence, autonomy, and relatedness (Ryan and Deci, 2017; Sweet et al., 2012). Competence is the feeling of effectiveness within one’s environment, which includes the ability to understand and process relevant information that is contextually and situationally applied (Ryan and Deci, 2017). This intrinsic process that enables competence can be elicited by an external stimulus, which is where therapeutic interventions exist. Autonomy is the volitional feeling in one’s choices that becomes an expression of intrinsic motivation supported by personal locus of control (Ryan and Deci, 2017; Fazey and Fazey, 2001). Relatedness is feeling accepted by one’s social environment with a sense of belonging and responsiveness with and to others (Ryan and Deci, 2017; Ntoumanis et al., 2021). Self-regulation and behavioral change are linked by these constructs that implicate individuals as self-determined agents of their actions. Interestingly, autonomy and relatedness have been hypothesized as antecedents to competency and self-efficacy. Autonomy prompts feelings of competence, which plays a crucial role in transpiring self-determination to self-efficacy (Ryan and Deci, 2000; Emsza et al., 2016).

A patient’s thoughts, perceptions, emotions, and behaviors are influenced by their autonomy. Autonomous exploration of beliefs and values, when coherent and efficient, results in personal competence. The motivational process leading to an empowered and sustainable recovery is achieved through competence gained from conviction of personal beliefs and values that are underscored by self-efficacy and self-regulation (Aujoulat et al., 2007; Hart et al., 2003; Conger and Kanungo, 1988). The transition away from powerlessness harnesses self-efficacy and self-regulation to execute and maintain action, respectively.

### Patient empowerment—*defined*

This manuscript aimed to synthesize a foundational definition of patient empowerment that inspires future consensus research through potential Delphi studies, nominal-group techniques, or content meaning analysis. Consensus means agreement within an academic or professional community around the internal content for a specific term. The critical review of TA & PCC and comprehensive analysis of the outlined interchangeable concepts and personal contextual factors form the internal content and are consolidated to frame a definition of patient empowerment.

#### Empowerment defined

**Figure 2:**
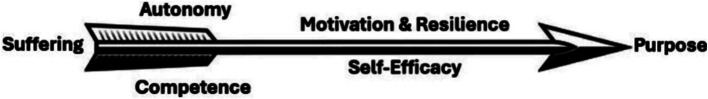
The arrow of empowerment: represents the potential energy stored with endured suffering and when released through acceptance is controlled by autonomy and competence; stabilized by motivation, resilience, and self-efficacy; and directed by purpose.

The journey of empowerment is co-created though individually directed toward an expected and sustainable outcome that is always slightly beyond reach signaling the moving target of human behavior and personal transformation. Empowerment is a complex multilayered behavioral transformation inspired by personal acceptance of and accountability to perception of powerlessness and suffering that transforms a renewed sense of purpose. The renewed sense of purpose endorsed by personal goals and values fuels self-determined behaviors. The transformation reflects a self-efficacious responsiveness gained through autonomy, competence, and relatedness with the reintegration of social resources. The transformation to empowerment is sustained by energy efficiency, intrinsic motivation, resilience, and self-regulation (see Figure 2).

## Discussion

A foundational theme for patient empowerment as a long-term solution for behavioral change and adaptations for self-management requires a restructuring of oneself. Insightful reflection of personal contextual factors enables a psychological experience toward personal transformation. Empowerment must be viewed as a transformative process built on the validation of the patient’s initial sense of powerlessness and associated contextual factors (Aujoulat et al., 2008). The purpose of this review was to highlight specific personal contextual factors that help forge an applicable definition of empowerment. The specific factors reviewed include suffering, meaning & purpose, motivation, resilience, energy-efficiency, and self-efficacy. These personal contextual factors are associated with internal control and external resources required for self-determined goals. The authors argue that these specific factors are critical variables for a full realization of patient empowerment. The argument is sustained by the recognition that TA and PCC have not fully explored these contextual factors. TA and PCC that validate these variables through empathic collaborative communication builds an essential trust required to assist the transformation. Authentic communication with emotional engagement harnesses active patient participation and serves as an antecedent to empowerment. The consequence of active patient participation with co-creative knowledge is autonomy, competence, relatedness, and intrinsic motivation. Autonomy, competence, and relatedness are antecedents to self-efficacy. These antecedents lead to behavioral change, which harnesses self-efficacy to execute action and self-regulation to maintain that action. Further, self-efficacy, self-mastery, and energy efficiency reflect attributes and consequences of successful patient empowerment. These interchangeable concepts and personal contextual factors offer a strategic application, support a comprehensive understanding, and frame the need for a consensus definition of empowerment for healthcare providers in rehabilitation settings.

The traditional healthcare model centered around passive delivery of interventions with expected compliance is perpetuated in many ways. Established research identifies how patient empowerment tends to address two issues: relating to healthcare providers and managing interventions (Aujoulat et al., 2008; Fugelli, 2001). This assertion aligns empowerment with PCC and TA as a compliance-oriented approach. More contemporary research argues that PPC driven by data collection and information gathering has aligned with the traditional biomedical model (Akseer et al., 2021; Aujoulat, 2024; Gibson et al., 2021). The evolution of PCC and TA paradigms to better involve patients falls short of advancing effective self-management (Duarte-Diaz et al., 2023). For example, a 2020 qualitative survey study of physiotherapists unites these concerns for the provisions of self-management by identifying that most respondents indicated PCC entailed working from the patient’s request for help and adapting their approach to the specifics of the request (Hutting et al., 2020). PCC and TA paradigms lack strategy for behavioral change without validation of a patient’s contextual factors and regulation capacity that contribute to empowerment.

To be fair, current literature provides notable progress describing effective PCC and TA that includes patient contextual factors (Hutting et al., 2022; Caneiro et al., 2021; Small et al., 2013). In Dahlberg et al. (2009) asserted empowerment requires understanding patient’s view on healthspan, lifespan, and well-being while fully embracing authentic principles of care consistent with compassion (Small et al., 2013). Additionally, Caneiro et al. (2021) offered comprehensive guiding principles for behavioral change that encouraged providers to consistently explore their patients’ emotional responses, explicit views, and implicit beliefs about their pain problems. Most recently, Hutting et al. (2019) outlined essential elements of PCC that incorporates a biopsychosocial understanding of the patient’s experience, person-centered communication, and support for self-management. Overall, these studies indicate that theoretical applications for empowerment and self-management premised on TA and PCC are declining. These studies seemingly support the argument in this review, a consensus on interchangeable concepts for empowerment and self-management require patient contextual factors for emotional engagement, which would serve the intentional strategy for the practical outlines. In the end, specifics with provisional strategies to satisfy the transformation toward empowerment were not fully satisfied.

Authentic TA is considered an antecedent and mediator to patient empowerment. TA “arises from a deep-rooted conviction that the patient is not a subordinate biomachine, but a fellow human being whom we should approach with humility, respect, and non-dominance.”(Tilden et al., 2005, p. 577) This personal understanding requires that empowerment must be situated by and with the patients themselves (Adams et al., 1997). Mora et al. (2022) discovered a significant effect between empowerment and patient factors, specifically quality of life, health status, self-efficacy, self-esteem, stigma, social support, and psychosocial symptoms. These findings underscore how TA and PCC must explore the contextual factors that reciprocate with empowerment. Specific interventions that forge empowerment premised on patient contextual factors require an exploration of self with the acceptance of suffering and the reinforcement of autonomy, competence, intrinsic motivation, self-efficacy and self-regulation toward purposeful gains (Caston et al., 2024; Duarte-Diaz et al., 2023; Hutting et al., 2019).

Small et al. (2013) qualitatively discovered five (5) dimensions of empowerment; identity, knowledge and understanding, personal control, personal decision-making, and enabling other patients with long term conditions. Small et al.’s (2013) interviews found empowerment developed through changes in perception of the self that minimized the illness experience through an effective communication to a basic level of understanding, through personal control of perceptions while developing strategies to overcome illness, through self-efficacy in personal decision-making preferences, and through empathic experiences that elevate awareness of others suffering with intent to motivate them to be persistent (Small et al., 2013). This qualitative description partially emulates the transformative process of empowerment. The Small study could be the closest evidence discovered that expressed the dynamic process of restructuring one’s experience with chronic illness, pain, or disability. The qualitative insights highlight the suggestion that empowerment requires a personal acceptance, a restructuring of self and perception, and a recognition of personal values that transpire into meaning making.

Other than the Small et al. study, the literature reviewed could not find clear evidence for how to operationalize the complexity of patient empowerment that facilitates a dynamic restructuring of patients’ identity with chronic illness, pain, and disabilities. The inability to operationalize empowerment may rest on the lacking consensus to define the term. Additionally, the overlapping utilization of interchangeable terms such as motivation, resilience, and self-efficacy obfuscates attempts to operationalize empowerment. The authors offered clear distinctions for those specific terms while maintaining alignment with empowerment. Likewise, the review expanded on patient contextual factors, such as suffering, purpose, and energy efficiency that fully merge PCC and TA with successful empowerment. The totality of these interchangeable concepts and patient contextual factors envelops the process of developing and maintaining empowerment.

Several investigations suggest distinct behaviors reflect empowerment. The literature review identified empowerment occurs by differentiating oneself from their illness or pain (Anderson and Funnell, 2010; Aujoulat et al., 2008; Tilden et al., 2005), by reintegrating a social identity and resources (Adams et al., 1997), by adjusting through acceptance and lowering expectations of full recovery (Edwards et al., 2016; Heidrich and Ward, 1992), and finding a way of meaning making by transforming one’s impact (Galletta et al., 2019; Mathieson and Stam, 1995; Nochi, 2000; Shapiro, 2005). These reflections of empowerment center on acceptance of personal suffering and changes in perception. Meaning and purpose are reconciled when an individual removes the discrepancy between their current identity and the identity they prefer. A reconciled self-identity is a part of coping that provides a sense of coherence and enables resilience (Galletta et al., 2019). A metamorphosis of identity by accepting the reality of the current state of existence leverages their experience toward empowerment.

A review of suffering is offered as an experiential context associated with the loss of identity and personal integrity, which must be accepted to alter the mind set associated with meaninglessness and emptiness. Acceptance provides patients with the paradoxical mechanism to gain control of their suffering and turn it into the fuel required to escape the gravitational pull of an identity bound to a diagnosis, disease, and dysfunction (Edwards et al., 2016). Empowerment is initiated and develops through the exploration of suffering that re-establishes personal meaning and purposeful direction grounded in self-identified values, goals, and resources.

**Figure 3:**
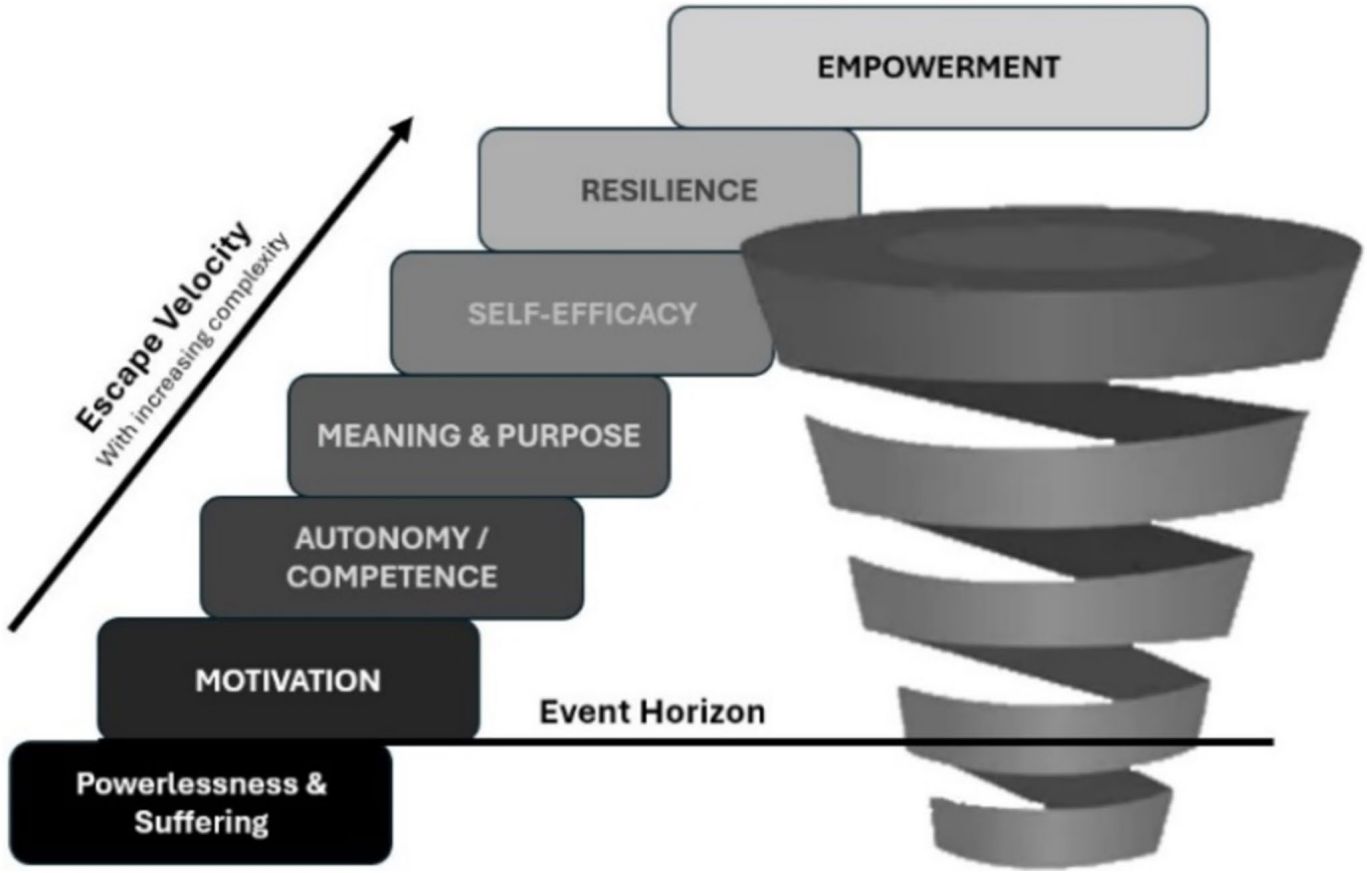
Escape velocity: represents Empowerment as the theoretical force established through motivation, autonomy, competence, meaning, purpose, self-efficacy, and resilience required to break the gravitational pull of powerlessness and suffering, which is also known as the Event Horizon. The break from the gravitational pull is directed by purpose.

The experience of suffering like depression narrows focus and the cognitive ability to process positive and regulate negative information (Varela and Melvin, 2023). The cognitive discrepancies were previously described as alignment of oneself with illness or pain, perceived disenfranchisement of identity and resources, an inability to accept current circumstance, and lacking any personal meaning or sense of purpose. This cognitive discrepancy and narrowed focus are the gravitational pull of powerlessness. Disempowerment reinforces the gravitational pull resulting in a downward spiral that can known as an Event Horizon. The trajectory of empowerment has a point where momentum allows an alignment of affective and cognitive pathways that ultimately supply the motivation and energy toward an Escape Velocity required to break the Event Horizon, the inescapable gravitational pull of cognitive discrepancies. It is conceivable to witness empowerment as the experience that moves above and below the Event Horizon. The fluctuations of empowerment further reinforce TA that consistently promotes self-exploration toward self-determination, self-efficacy, and self-regulation. These characteristics are vital to achieving Escape Velocity and sustaining empowerment. Moreover, this higher order thinking associated with self-efficacy, self-regulation, and ultimate self-mastery are fundamental coping and managing skills for patients to escape the gravitational pull of powerlessness (see Figure 3).

## Conclusion

The science of human behavior has escaped healthcare’s consideration of empowerment as a transformation. Global health burdens would benefit from behavioral interventions to address increasing levels of chronic disease, dysfunction, pain, and disability. Yet, patients are blamed for the insufficient outcomes though many are considered dependent on the healthcare system. The challenges to improve outcomes are further marked by multiple definitions for patient empowerment that are inconsistently used. More importantly, the empirical application for patient empowerment seemingly overlooks interventional mechanisms, processes, and strategies. The authors argued that specific interchangeable concepts and personal contextual factors are variables that act as confounders and modifiers for an applicable definition of patient empowerment and offers potential strategies to empower patients. The provider-patient relationship within the dynamics of the healthcare system unites these overlapping concepts through TA and PCC built on collaborative communication and trust. The aim of this manuscript was to support a comprehensive and contextual understanding of empowerment and frame the need for a consensus definition for healthcare providers in rehabilitation settings. A foundational definition was synthesized through theoretical reviews of the specific concepts and factors. The interchangeable concepts and contextual patient factors that potentially modify successful recovery and sustainable empowerment remain unclear and require further consensus agreement and investigation.

## Data Availability

The original contributions presented in the study are included in the article/supplementary material, further inquiries can be directed to the corresponding author.
